# The Use of Tutoplast as an Adjunct in Scleral Buckle Procedure in Patients with Extremely Thin Sclera

**DOI:** 10.18502/jovr.v16i3.9448

**Published:** 2021-07-29

**Authors:** Matthew R. Starr, Sophie J. Bakri

**Affiliations:** ^1^Department of Ophthalmology, Mayo Clinic, Rochester, MN, USA

**Keywords:** Retinal Detachment, Scleral Buckle, Scleral Thinning, Scleromalacia

## Abstract

Extremely thin sclera often necessitates abortion of scleral buckle procedures. In patients in whom a scleral buckle is desired, previous techniques have included the use of cyanoacrylate glue and continuing with surgery or placing donor tissue over the areas of thin sclera, but this can delay surgery. This was a retrospective review of three patients with thin sclera encountered during scleral buckling procedures. All patients had Tutoplast Pericardial Graft placed over the areas of thin sclera which allowed the scleral buckle to be sutured onto the Tutoplast rather than the thin sclera. Tutoplast Pericardial Graft is a useful adjunct in scleral buckle procedures with extremely thin sclera, and a scleral buckle can be safely placed over it and lead to successful retinal reattachment.

##  INTRODUCTION

Encountering thin sclera during retinal detachment surgery can pose a significant problem, particularly when a scleral buckle is to be performed. Sternberg and colleagues pioneered the use of sutureless scleral buckling in 1988 with the use of cyanoacrylate glue.^[1]^ Previous reports had glued the buckle directly onto the sclera which did not allow the surgeons to adjust the buckle. Sternberg et al described fixing a series of plates with belt loops to the sclera using cyanoacrylate glue with which the buckle could be threaded through and then further adjusted. Other reports have discussed the use of donor scleral tissue to repair thin sclera, but delaying retinal detachment surgery.^[2]^ We present three patients who during retinal detachment surgery were noted to have extremely thin sclera. Rather than avoiding a scleral buckle, Tutoplast Pericardial Graft (Katena Products Inc., Denville, NJ) was used to reinforce the sclera and continue with the scleral buckle procedure. Tutoplast is a dehydrated, processed pericardium from human donor tissue that is converted into a multidirectional, collagen tissue matrix with a thickness of 400 microns.^[3]^ It is meant to use for the repair, replacement, reconstruction, or augmentation of soft tissue. This research complied with HIPAA and the Declarations of Helsinki. Consent to publish these findings and images were gathered from the patients.

**Figure 1:**
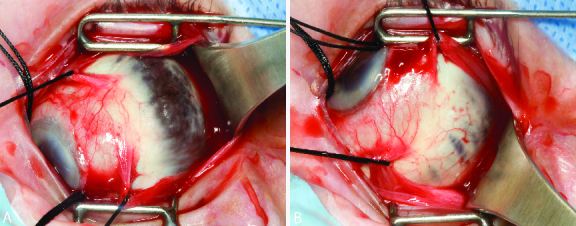
Intraoperative external photograph of an 80-year-old female during placement of a scleral buckle showing extensive scleral thinning in the superotemporal quadrant (A). Additional scleral thinning was noted inferotemporally as well (B).

**Figure 2:**
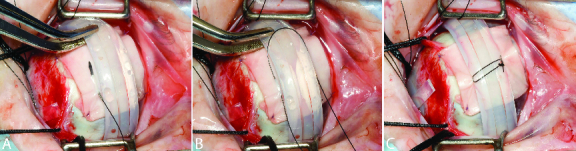
Intraoperative external photograph of the same patient in Figure 1 showing Tutoplast covering the extensive scleral thinning superotemporally with 7-0 Vicryl sutures tacking the Tutoplast to thicker sclera at each corner (A). A horizontal mattress suture using 5-0 nylon is then placed within the Tutoplast and not in the sclera itself (B). The suture is then tied around the scleral buckle (A–C) and the knot is rotated inferiorly (C).

##  SURGICAL METHOD

For all three patients, a conjunctival peritomy was performed 360º at the limbus and bluntly dissected down to bare sclera. The tenon was then bluntly dissected in all four quadrants. The four rectus muscles were then looped and isolated using 2-0 silk suture. The scleral quadrants were inspected, and there was found to be extensive scleral thinning in one or more quadrants in all three eyes [Figures 1A and 1B]. In all patients, Tutoplast Pericardial Graft was placed over the areas of thin sclera in order to facilitate the placement of the scleral buckle. The size of the Tutoplast was adjusted in order to be large enough to avoid passing a suture through the thin sclera. The Tutoplast was sutured onto the sclera with one 7-0 Vicryl suture in each of the corners with the sutures placed where the sclera was noticeably thicker [Figure 2A]. A 287 scleral buckle with a 240 band was then looped underneath the four rectus muscles and over the Tutoplast. Subsequently, a 5-0 nylon suture was placed in a mattress fashion within the Tutoplast with the sutures 9 mm apart [Figures 2A–2C]. The nylon sutures were tied and the knot was rotated posteriorly. The remainder of the case was completed as normal and the tenon and conjunctival layers were closed over the Tutoplast at the limbus without difficulty in all three patients.

##  RESULTS

All three patients had concomitant vitrectomies at the time of the scleral buckle surgery with the use of endolaser with no cryotherapy performed. One patient detached one month following the scleral buckle, due to the development of proliferative vitreoretinopathy but then was successfully reattached with silicone oil (six years of follow-up) while the other two remained attached at the time of the last follow-up visit (three years of follow-up for both patients).

##  DISCUSSION

Tutoplast is a viable alternative to donor tissue or glue when thin sclera is encountered during scleral buckling surgery. Tutoplast can be readily stored in operating rooms and has a five year shelf life.^[3]^ Certainly, other procedures for retinal detachment repair such as a primary pars plana vitrectomy should be considered when thin sclera is encountered, but if a buckle is felt to be absolutely needed, Tutoplast can be applied over the areas of thin sclera and offer support for the scleral buckle. As outlined by Sternberg and colleagues, the use of cyanoacrylate adhesive is an additional means for affixing a scleral buckle to the sclera without the use of sutures or scleral tunnels.^[1]^ Certainly, this method may provide immediate adhesion, but long-term buckle placement may be a potential downfall. With the use of Tutoplast, the patch graft is anchored to the sclera with sutures and then the buckle sutures are placed into the Tutoplast itself, offering long-term viability. Other sutureless buckling methods such as scleral tunnels are also not possible in patients with advanced scleromalacia due to the persistent perforation risk. Finally, if only a single quadrant of sclera is thin, the use of sutures or tunnels in a triangular pattern for three-point fixation, rather than four-point, is possible, and can provide appropriate buckle support. In our series, one patient had already failed primary vitrectomy and thus was felt to require scleral buckle placement, while the other two had inferior pathology which was felt to be best supported with buckle placement in addition to pars plana vitrectomy and thus the decision was made to proceed with Tutoplast-assisted scleral buckling.

##  Financial Support and Sponsorship

Nil.

##  Conflicts of Interest

There are no conflicts of interest.

Ethics approval: IRB approval waived.

Consent to participate: Consent to publish these findings and images were gathered from the patients.
